# Superhydrophobic Surface Based on a Coral-Like Hierarchical Structure of ZnO

**DOI:** 10.1371/journal.pone.0014475

**Published:** 2010-12-30

**Authors:** Jun Wu, Jun Xia, Wei Lei, Baoping Wang

**Affiliations:** School of Electronic Science and Engineering, Southeast University, Nanjing, People's Republic of China; Massachusetts Institute of Technology, United States of America

## Abstract

**Background:**

Fabrication of superhydrophobic surfaces has attracted much interest in the past decade. The fabrication methods that have been studied are chemical vapour deposition, the sol-gel method, etching technique, electrochemical deposition, the layer-by-layer deposition, and so on. Simple and inexpensive methods for manufacturing environmentally stable superhydrophobic surfaces have also been proposed lately. However, work referring to the influence of special structures on the wettability, such as hierarchical ZnO nanostructures, is rare.

**Methodology:**

This study presents a simple and reproducible method to fabricate a superhydrophobic surface with micro-scale roughness based on zinc oxide (ZnO) hierarchical structure, which is grown by the hydrothermal method with an alkaline aqueous solution. Coral-like structures of ZnO were fabricated on a glass substrate with a micro-scale roughness, while the antennas of the coral formed the nano-scale roughness. The fresh ZnO films exhibited excellent superhydrophilicity (the apparent contact angle for water droplet was about 0°), while the ability to be wet could be changed to superhydrophobicity after spin-coating Teflon (the apparent contact angle greater than 168°). The procedure reported here can be applied to substrates consisting of other materials and having various shapes.

**Results:**

The new process is convenient and environmentally friendly compared to conventional methods. Furthermore, the hierarchical structure generates the extraordinary solid/gas/liquid three-phase contact interface, which is the essential characteristic for a superhydrophobic surface.

## Introduction

The lotus leaves-like surface, which has a high apparent contact angle, displays perfect water repelling and self-cleaning properties and is referred to the superhydrophobicity [1]. In the past decade, inspired by the superhydrophobic properties of such surfaces, many research groups have studied the fabrication of superhydrophobic surfaces because of the potential applications in areas such as micro-fluidics, self-cleaning surfaces, lab-on-chip devices, low friction coatings, water proof and hydrophobic textiles, etc [2]–[14].

It is well known that the superhydrophobicity is governed by both the chemical composition and the geometrical structure of the solid surface [15]–[21]. As a result, a significant amount of effort was devoted to the construction of structures with a roughness at both micro- and nano-meter scales. Some examples of these methods include chemical vapour deposition [22], the sol-gel method [23], etching technique [24], electrochemical deposition [25], the layer-by-layer deposition method [26], etc. However, most of these methods are complicated, costly, and have a drawback of poor environmental stability from the viewpoint of practical applications. Recently, some robust methods allowing manufacturing of stable superhydrophobic surfaces have been reported [27]–[28]. ZnO films have attracted great attention due to its excellent chemical and thermal stability [29]–[30]. Recently, there are also some reports about ZnO films with superhydrophobic properties. However, most of these studies works refer to one-dimensional nanomaterials (nanowires or nanorods) [31]–[33]. Limited studies have been performed on the superhydrophobicity on lotus leaves like ZnO surfaces. Kim et al fabricated dual-scaled surfaces by the combination of a conventional silicon wet-etching with a ZnO solution method [34]; Jun Wang et al also have successfully fabricated a hierarchical ZnO structure on a sapphire substrate [35]. But the methods mentioned in their articles show several shortcomings: (1) high control temperature; (2) expensive infrequent materials; (3) complicated and time consuming processing routes.

In this paper, we report a simple, reproducible method to fabricate superhydrophobic surface with hierarchical structure based on ZnO nanorods generated by an alkaline hydrothermal method. The wetting property of our ZnO film was characterized before and after treatment with Teflon. In our case, the high roughness ZnO surface generated a perfect superhydrophilicity, and the superhydrophobicity behaviour is achieved by the hierarchical structured ZnO in combination with the low surface energy coating of Teflon.

## Results and Discussion

**Figure 1:**
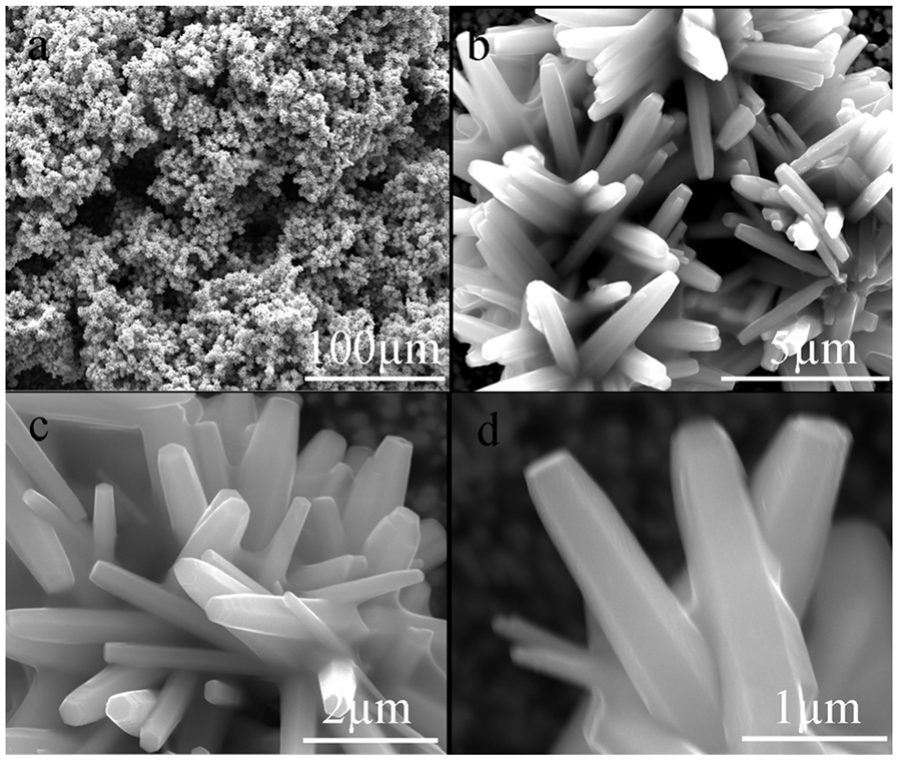
FE-SEM images of the ZnO films constructed on glass substrate with a magnification of (a) ×800, (b) ×20000, (c) ×40000, (d) ×80000.

The morphology of ZnO film formed on glass was investigated by FE-SEM. Figure 1a is a top view of a coral-like microscopic structure, which enhances the surface roughness. The details of the coral can also be clearly observed in Figure 1b. This enlarged view of the coral shows that they are built from quasi-oriented antennas (Figure 1c), which shows that the antenna has a width of about 0.5–1 µm. This observation indicates that the hierarchical structure is formed on the glass substrate. The corals with diameter of about 12 µm distributing on the glass generate the micro-scale roughness. The nano-scale roughness is determined by the antennas of the corals. It is well known that the growth of one-dimensional ZnO nanorods is easy; however a hierarchical structured surface generated by one step method is unusual. In our present experiment, hydrothermal method has provided a convenient way to fabricate the hierarchical structured surface. It is interesting to mention that there is no strict requirement on the materials and substrate shape in a hydrothermal method; so, this coral-like ZnO films can be synthesized on substrates consisting of other materials and having various shapes.

**Figure 2:**
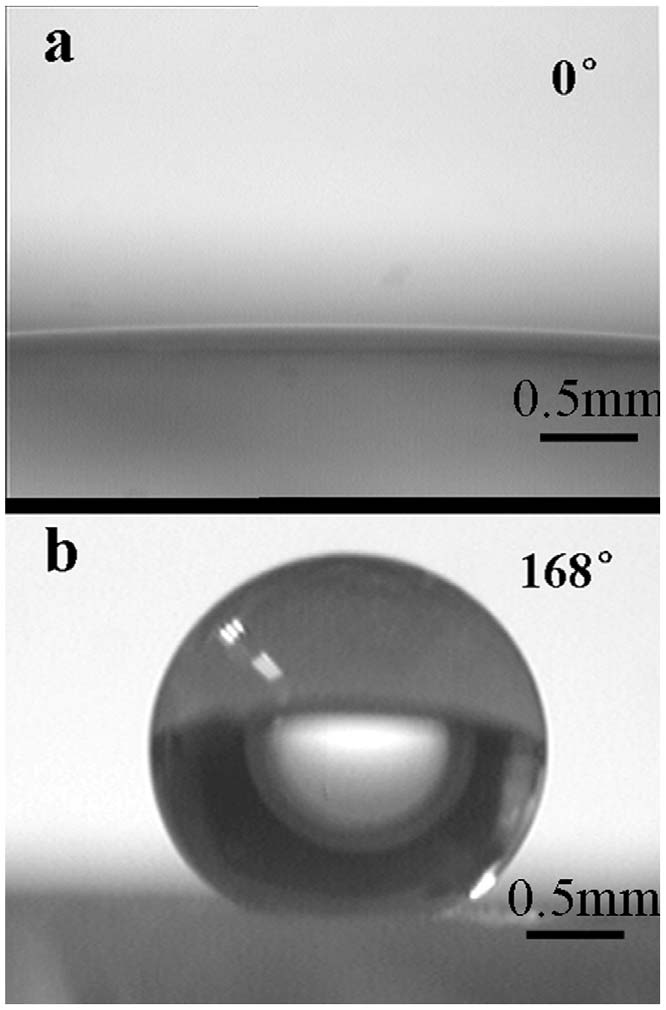
Different water droplet configurations. (a) Water droplet spread on the superhydrophilic ZnO film, and (b) a water bead standing on a Teflon-modified ZnO surface.



As mentioned above, the original ZnO film is superhydrophilic with an apparent contact angle near to 0° (Figure 2a). A water droplet spreads immediately when it is placed on the as-prepared ZnO substrate. The apparent contact angle value is unchanged even after keeping the sample in air for 1 h. The stability of such a superhydrophilic surface has been proved repeatedly and reproducibly. This superhydrophilic phenomenon comes from the high surface energy of the crystals which are generated in aqueous condition. After roughening such a material with a hierarchical structure, a superhydrophilic surface is obtained of which the degree of hydrophilicity is determined by the proportion of the geometric projected area to the actual area. As described by Wenzel's theory, the apparent contact angle used to describe the wettability is given by equation (1):

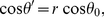
(1)where 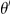 and 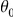 are the apparent contact angles on solids with rough and smooth surfaces, respectively, *r* is the roughness factor, defined as the ratio of the actual area of a rough surface to the geometric projected area. For a rough hydrophilic surface, the liquid is in full contact with the solid surface. As we can see, for a hydrophilic surface with extremely high roughness factor, the cosine value of 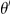 can be as big as 1, which means that an apparent contact angle of almost 0° is observed on such a surface.

To fabricate a superhydrophobic surface, Teflon is used to tune the surface wettability of the ZnO film. After modification with Teflon, the ZnO film changes its wettability to superhydrophobicity. The shape of a water droplet with 3 µL on the ZnO film is spherical with an apparent contact angle about 168°, just as shown in Figure 2b. The sliding angle is less than 5°, measured by inclining the flat sheet degree by degree. Both the apparent contact angle and sliding angle indicate the superhydrophobicity of the ZnO film. To understand the superhydrophobicity of the ZnO films, a mathematical model based on the Cassie-Baxter hypothesis of air trapping under a water drop is utilized [28]. As introduced in the model, the presence of double scale roughness itself is not a sufficient condition for obtaining the good superhydrophobicity. In our case, the air-water interface is not only provided by the space between the coral-like microscopic structures but also supplied by the gaps between the antennas. As a result, the large air-water interface fraction induced a high apparent contact angle.

We found a simple, repeatable method to fabricate a ZnO hierarchical structured surface. Micro-scale roughness was generated by ZnO corals with a width of 10 µm built on the substrate; nano-scale roughness was formed by the antennas. The prepared ZnO film exhibited a superhydrophilic trait with an apparent contact angle around 0°. A superhydrophobic surface, i.e. an apparent contact angle around 168°, was obtained by surface modification with Teflon. The whole procedure reported here is convenient, environmental-friendly compared to the conventional methods. Our new way of roughening a glass surface can be applied also to other substrates such as metals or polymers.

## Methods

The ZnO coral film was prepared in an alkaline aqueous solution at hydrothermal condition, and the morphology of the obtained ZnO coral-like structures were observed by Field Emission Scanning Electron Microscope (FE-SEM). The fabrication of the ZnO coral film was carried out as follows. A flat glass sheet being the substrate for sample growing was ultrasonically washed with acetone, ethanol and deionised water, respectively. A simple and effective spraying method to plant the ZnO seed layer on the substrate is utilized at 300°C. The solution used to spray is a Zn-AD (Zn(CH_3_COO)_2_ 2H_2_O) with the concentration of 0.5 M for half an hour. Then, the seeds-coated glass substrate was cooled in air. Finally, the substrate was placed in bottles containing the ZnO coral growth solution, being 0.15 M Zn-AD. The hydrothermal condition used to heat the reactive systems was done in a water tank for 6 hours at 95°C. The substrate was dried in a drying oven at 100°C for half an hour and subsequently they were rinsed in deionised water for 3 minutes to remove any residual dust particles from their interspaces.

The wettability of the sample was tested by a water droplet with a volume of 3 µL in an environmental chamber under ambient condition (25°C). The apparent contact angle formed on this hierarchical structured film was very low, down to ∼0°. After been modified by Teflon-AF (DuPont, USA) using spin-coating, the sample exhibited perfect superhydrophobicity, which was demonstrated by the apparent contact angle of water droplet of the same volume. Five different spots on the surface were tested to verify the uniformity (which were 168.2°, 168.3°, 167.1°, 167.9°, and 167.5°). The adopted data corresponded to the stable mean value of the angle. The uncertainties in the measurements were within ±2°.
